# To Be or Not to Be: Circular RNAs or mRNAs From Circular DNAs?

**DOI:** 10.3389/fgene.2019.00940

**Published:** 2019-10-11

**Authors:** Leire Iparraguirre, Iñigo Prada-Luengo, Birgitte Regenberg, David Otaegui

**Affiliations:** ^1^Neurosciences Area, Biodonostia Health Research Institute, San Sebastián, Spain; ^2^Department of Biology, University of Copenhagen, Copenhagen, Denmark

**Keywords:** circular RNA, mRNAs, NGS, back-splacing junctions, Circular DNAs

## Abstract

In recent years, there has been a growing interest in circular RNAs (circRNAs) since they are involved in a wide spectrum of cellular functions that might have a large impact on phenotype and disease. CircRNAs are mainly recorded by RNA-Seq and computational methods focused on the detection of back-splicing junction sequences considered the diagnostic feature of circRNAs. While some protocols remove linear RNA prior to sequencing, many have characterized circRNAs by sorting through total RNA sequencing data without excluding the possibility that some linear RNA can provide the same signal as a circRNA. Recent studies have revealed that circular DNAs of chromosomal origin are common in eukaryotic genomes and that they can be transcribed. Transcription events across the junction of circular DNAs would result in a transcript with a junction similar to those present in circRNAs. Therefore, in this report, we want to draw attention to transcripts from such circular DNAs both as an interesting new player in the transcriptome and also as a confounding factor that must be taken into account when studying circRNAs.

## Significance

There is a growing interest in circRNAs due to their implication in many biological processes and diseases in addition to their biomarker potential. They are mainly detected by the presence of reads mapping their backsplicing junction. Nevertheless, circRNAs are no longer the only transcripts containing such a junction since recent studies have revealed that circular DNAs are common and can be transcribed resulting in transcripts that would mimic a circRNA signal. Therefore, this new type of chimeric transcript can change the way in which circRNA analysis is being done and impact some of the results already reported.

## Are Circular RNAs the Only Chimeric Transcripts?

**Figure 1:**
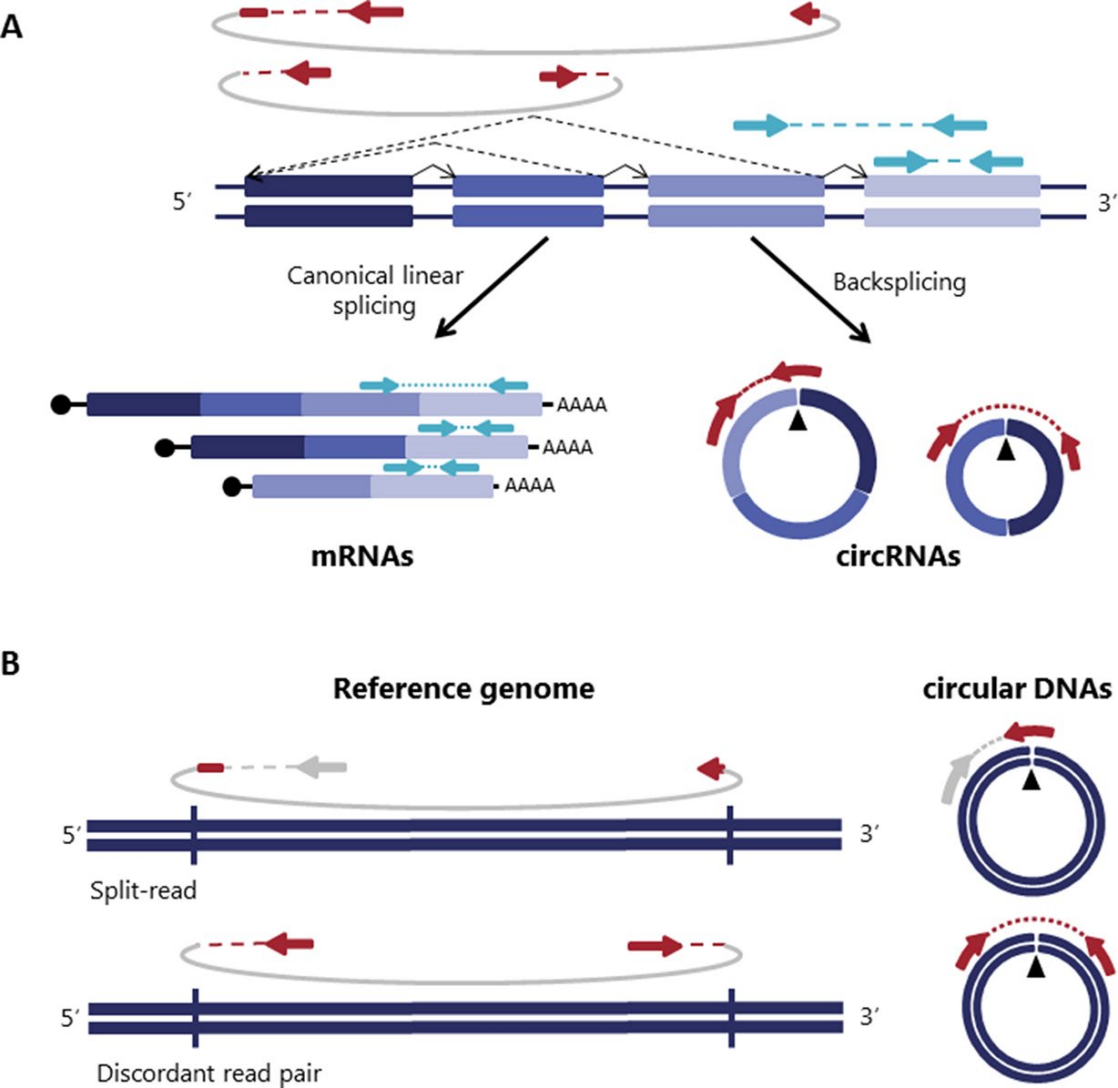
Detection of both circular RNA and DNAs is based on reads spanning the junction. **(A)** Backspliced joining between exons and canonical exon joining are depicted with non-continuous and continuous lines respectively giving rise to circRNAs and mRNAs. Paired-end reads spanning the backspliced junctions are shown in red and paired-end reads consistent with a linear transcript are shown in blue. **(B)** Similar to circRNAs, circular DNAs (eccDNAs) are detected based on structural-read variants consistent with a circularization event depicted in red [adapted from (Møller et al., 2018)].

Circular RNA (circRNAs) were rediscovered a few years ago as non-canonically spliced RNA forms present in different organisms including humans (Salzman et al., 2012; Jeck et al., 2013). They are covalently closed transcripts formed through an RNA back-splicing event, where a splice donor of a downstream exon joins to an upstream splice acceptor leading to covalently closed transcripts that are characterized by the presence of a back-splicing junction that makes circRNAs distinguishable from their linear counterparts (**Figure 1A**) (Zhang et al., 2016; Wilusz, 2018).

Since their rediscovery, the scientific community has drawn its attention to circRNAs and has investigated their involvement in several cellular processes in health and disease (Haque and Harries, 2017), their potential role as biomarkers (Abu and Jamal, 2016), and their regulatory functions (Floris et al., 2016). CircRNAs are now known to be abundant and stable in the cytosol and the nucleus (Salzman et al., 2012; Jeck et al., 2013; Li et al., 2015) and have also been found free in biofluids (Bahn et al., 2015; Memczak et al., 2015; Chen et al., 2018) and in extracellular vesicles (Kyoung Mi et al., 2017). The biomarker potential of circRNAs has been intensely studied, in fact, there have been published many case-control studies seeking for differentially expressed circRNAs that could be biomarkers of different diseases. To date, circRNAs have been implicated in several diseases including cancer (Kristensen et al., 2017; Arnaiz et al., 2018), neurological disorders (Akhter, 2018), cardiovascular diseases (Aufiero et al., 2019) and immune-related diseases (Iparraguirre et al., 2017; Liu et al., 2019). At the same time, getting to fully understand their biogenesis, characteristics, functions, and implications in human biology remain as open questions for researchers in the field.

Although the function of most of the circRNAs remains unknown, it has been shown that some circRNAs can act as microRNA sponges, regulating the microRNA levels and their activity (Hansen et al., 2013; Memczak et al., 2013; Zheng et al., 2016). They are involved in gene expression regulation by regulating the transcription of their parental genes, competing with the linear splicing or sponging proteins (Ashwal-Fluss et al., 2014; Li et al., 2018). Interestingly, ribosome profiling studies have recently shown that circRNAs can also be translated both *in vitro* and *in vivo* (Legnini et al., 2017; Yang et al., 2017).

The main feature of circRNAs, and responsible for most of their special properties, is their circularity. Therefore, besides detecting their characteristic back-spliced junction, testing the circularity of these molecules, is one of the key points in every circRNA study. Nevertheless, many studies have based their discovery of circRNAs on total RNA and might thereby have interpreted some linear chimeric transcripts as circRNAs, resulting in false positive circRNA detections. To circumvent this problem most studies have confirmed the circularity of the transcripts found by total RNA-seq using RNase R, Northern blot or electrophoretic methods (Jeck and Sharpless, 2014). However, these circularity validations have also sometimes revealed transcripts that seem to be linear, rather than circular confirming that the detection of circRNAs starting from total RNA can lead to some false positives. These false positives have been attributed to technical artifacts or transcripts derived from uncommon events such as exon duplications or transplicing events (Jeck and Sharpless, 2014; Szabo and Salzman, 2016). That said, the option of having found true, biologically active and functional linear transcripts that contain a sequence equivalent to a backsplicing junction (from now on called chimeric linear transcripts), has been somewhat overlooked because a source of such linear RNA has not been known for healthy cells.

## Circular DNAs as a Source of Chimeric Linear Transcripts

Most of the human genome is organized in linear chromosomes, however, some exceptions have long been accepted such as mitochondrial DNA, and chromosomal aberrations such as DNA circles carrying oncogenes (e.g. double minutes) (Benner et al., 1991; Nathanson et al., 2014; Turner et al., 2017) and ring chromosomes (Tümer et al., 2004). It was not until recently that different circular DNAs such as microDNAs (Shibata et al., 2012) or extrachromosomal circular DNAs (eccDNAs) were found to also arise from large parts of different eukaryotic genomes including human and yeast (Møller et al., 2015; Kumar et al., 2017; Møller et al., 2018).

Circular DNAs are formed when two ends of a linear DNA are joint together resulting in a junction similar to the backspliced junction on circRNAs commonly called breakpoint junction that is detected based on structural-read variants consistent with a circularization event (Gresham et al., 2010; Møller et al., 2018; Prada-Luengo et al., 2019) (**Figure 1B**). They usually range from a hundred bases to megabase circles and can contain full exons and genes (Shibata et al., 2012; Møller et al., 2015; Kumar et al., 2017; Turner et al., 2017; Møller et al., 2018) and while some regions of the genome are more commonly found on circular DNA (Sinclair and Guarente, 1997; ; Møller et al., 2016; Turner et al., 2017; Møller et al., 2018), most circular DNA appear to occur at random (Shibata et al., 2012; Møller et al., 2015; Kumar et al., 2017; Møller et al., 2018).

**Figure 2:**
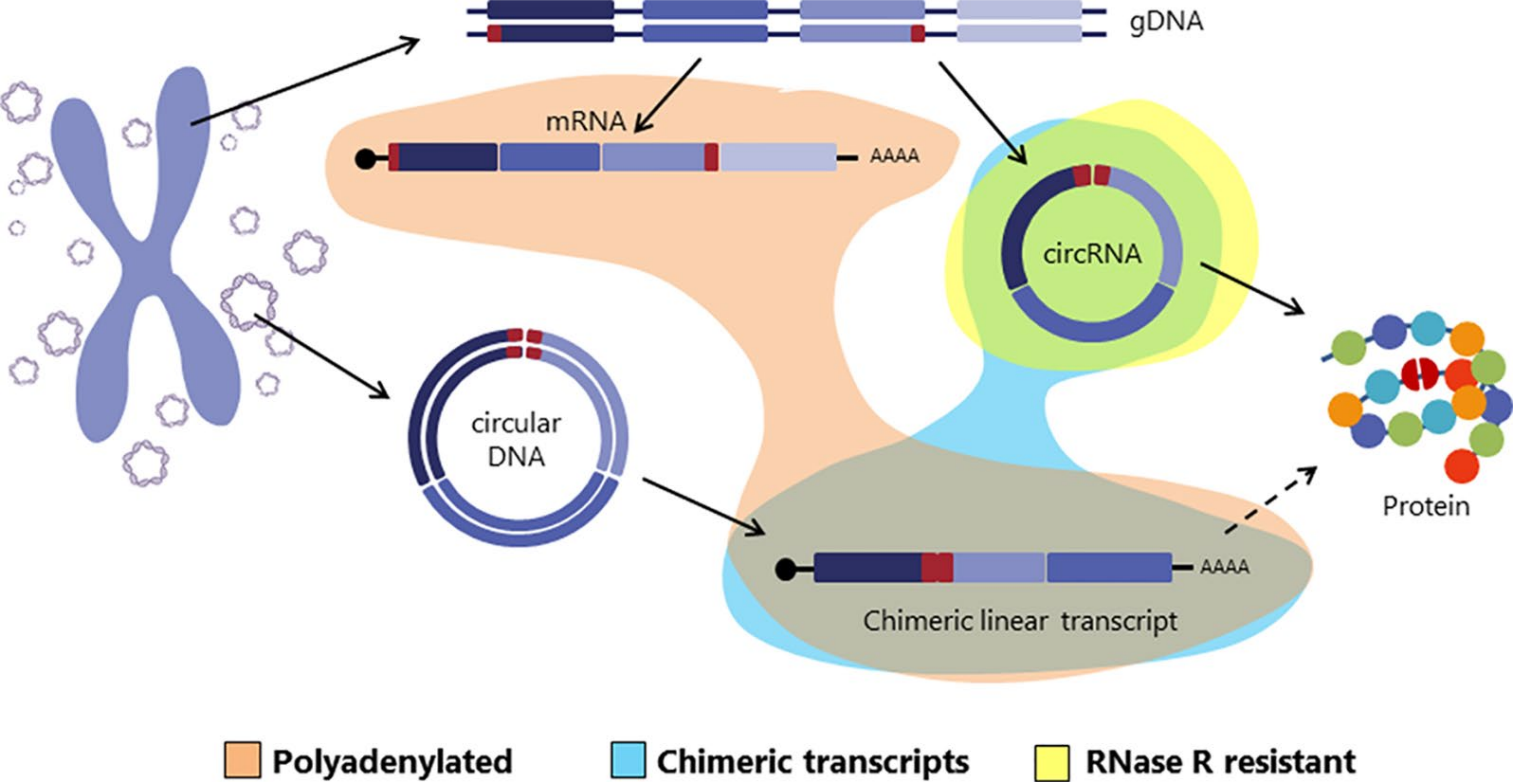
Graphical representation of different transcripts arised from either genomic DNA or circular DNA. Exons are colored in purple and the backspliced junctions or chimeric junctions are shown in red. Polyadenylated, chimeric junction containing and RNase R resistant transcripts are highlighted in orange, blue and yellow respectively.

Interestingly, in a recent paper Møller et al. identified thousands of eccDNAs in leucocytes and muscle cells in healthy controls. With the idea of investigating whether eccDNAs could be transcribed, an mRNA library was also sequenced from muscle tissue and analyzed for transcription events across the breakpoint junction of the detected eccDNA finding several matches (Møller et al., 2018). This finding suggests that circular DNA in healthy tissue is transcribed, giving rise to linear and polyadenylated transcripts that will carry a sequence equivalent to the backsplicing sequence of circRNAs (Møller et al., 2018) (**Figure 2**).

The transcriptional evidence of circular DNAs, together with their abundance, lead us to suggest that circular DNAs could be a natural source of a substantial amount of linear RNAs carrying chimeric junctions. In many cases, these chimeric junctions might be indistinguishable from the backsplicing junctions of circRNAs, and therefore they might be confounding factors in circRNA studies. In the following paragraphs, we will explain the data supporting this proposal.

## circRNA Detection: All That Glitters Is Not Gold

As previously introduced, circRNAs are formed though a non-canonical splicing event called backsplicing. Transcripts resulting from this backsplicing event have a covalently closed loop structure with neither 5′–3′ polarity, nor a polyadenylated tail and more importantly, they are characterized by the presence of a scrambled exon order relative to the linear transcript (Zhang et al., 2016; Wilusz, 2018). This scrambled exon order becomes evident in the backspliced junction that connects a 5’ downstream sequence with an upstream 3’ sequence. Thus, all the circRNA detection algorithms exploit the presence of the back-spliced junctions as a diagnostic feature for the identification of circRNA (**Figure 1A**).

Different methods have been adapted for the detection of these back-spliced junctions. Commercial arrays containing probes targeting these backspliced regions have been widely used in biomarker screening studies (Iparraguirre et al., 2017; Liu et al., 2017; Sui et al., 2017; Li et al., 2018). The subsequent validation is often also based on the amplification of the backsplicing junctions using divergent primers (Panda and Gorospe, 2018). Many other papers have conducted a high throughput sequencing analysis that overcomes one of the main limitations of the arrays allowing to detect not only the annotated circRNAs but also *de novo* RNA circularization events from genomic regions where no circRNA were annotated by previous studies. Several bioinformatic pipelines have been developed for the detection of circRNAs in RNA-Seq datasets, but all of them are based on the presence of reads crossing over the back-splicing junctions and finding the most reliable one is still a challenge for bioinformaticians (Hansen et al., 2016; Hansen, 2018; Prada-Luengo et al., 2019).

Two main approaches can be followed for the detection of circRNAs in RNA-Seq data. Firstly, many circRNA RNA-seq studies are based on RNase R treated samples in order to deplete all the linear RNAs before sequencing. Although this approach is specially designed for the circRNA detection it is worth noting that RNase R degradation is variable, that there are rare cases of RNase R resistant linear RNAs and RNase R sensitive circRNAs (Szabo and Salzman, 2016) and that this treatment requires a high RNA input which could be limiting for some tissues. Other circRNA studies choose to sequence either total, ribosomal-depleted (ribo-), or non-polyadenylated (polyA-) RNA, where both linear and circular RNAs can be found (Salzman et al., 2012; Memczak et al., 2013; Broadbent et al., 2015; Lu et al., 2015; Memczak et al., 2015). This approach avoids the use of RNase R, which reduces the RNA amount needed for the sequencing and allows studying the expression of other types of RNAs from the same dataset. It has been demonstrated that with a good sequencing depth and quality and a carefully data-analysis, true circRNAs can be detected from total RNA sequencing (Wang et al., 2017), however, in this second approach, a later circularity confirmation for circRNAs is needed.

With the discovery of linear chimeric RNAs transcribed from circular DNAs, circRNAs are no longer the only transcripts with chimeric junctions. Therefore, it is of utmost importance to note that whereas the first approach will significantly enrich the RNA sample in circRNAs so that most of the detected chimeric junctions will correspond to true circRNAs, the second one might overestimate the number of circRNA transcripts by attributing to circRNAs the signal coming from both circRNAs and the linear chimeric transcripts transcribed from circular DNAs. Consequently, taking into account the coexistence of circRNAs and linear chimeric transcripts, the need of circularity tests and functionality assays gains importance and special care should be taken regarding not only experimental but also computational methods to avoid mistaking chimeric transcripts from circular DNAs with circRNAs formed by backsplicing.

## Discussion

The circRNA field is still at an early stage, however, circRNAs have already shown to be astonishing molecules, implicated in many processes, with a great biomarker potential and that can also change the way we understand the transcription and translation processes. For these reasons, they are gaining attention and the circRNA field is at the moment one of the most active RNA research fields. However, there are still many conflicts, controversies and open questions (Li, 2019) that have to be discussed.

In this report, and in light of the recent advances in the circular DNA field, we want to point out the transcription from extrachromosomal circular DNA as one of the main natural sources of linear transcripts with back-spliced signals that could be interfering with circRNA data (Møller et al., 2018). From now on, apart from the technical artifacts, duplications and transplicing events that could lead to false positives in the circRNA detection, we should take also into account the existence of this new type of chimeric transcripts. Therefore circularization tests and functional assays are more important than ever.

In any case, these chimeric linear transcripts should not only be considered as a mere confounding factor for circRNA studies. Despite the technical implications for the circRNA characterization, the existence of these circRNA-like chimeric linear RNA molecules coming from eccDNAs adds a new type of molecule to the ever-growing list of RNAs and expands our vision about the complexity of the transcriptome and its regulation. Moreover, these linear RNA molecules coming from eccDNA could also present functions similar to the circRNA, including the regulatory functions or the potential to be translated. Gene products from eccDNA transcripts could potentially contribute to the phenotype of somatic cells and tissue as reported in yeast (Gresham et al., 2010; Demeke et al., 2015). However in this nascent field, more data and research is needed to start scratching the surface of the iceberg.

## Author Contributions

LI, IP-L, BR, and DO wrote the paper.

## Funding

This study has been funded by Instituto de Salud Carlos III through the project "PI17/00189" (Co-funded by European Regional Development Fund/European Social Fund) "Investing in your future"). IP-L and BR were supported by the Danish Council for Independent Research, 6108-00171B and LI was supported by the Department of Education of the Basque Government [grant number PRE_2018_2_0081].

## Conflict of Interest

The authors declare that the research was conducted in the absence of any commercial or financial relationships that could be construed as a potential conflict of interest.
